# Deep-ADCA: Development and Validation of Deep Learning Model for Automated Diagnosis Code Assignment Using Clinical Notes in Electronic Medical Records

**DOI:** 10.3390/jpm12050707

**Published:** 2022-04-28

**Authors:** Jakir Hossain Bhuiyan Masud, Chiang Shun, Chen-Cheng Kuo, Md. Mohaimenul Islam, Chih-Yang Yeh, Hsuan-Chia Yang, Ming-Chin Lin

**Affiliations:** 1 Graduate Institute of Biomedical Informatics, College of Medical Science and Technology, Taipei Medical University, Taipei 11031, Taiwan; d610105005@tmu.edu.tw (J.H.B.M.); 13507@s.tmu.edu.tw (C.S.); m610105009@tmu.edu.tw (C.-C.K.); d610104001@tmu.edu.tw (C.-Y.Y.); 2 Department of Otolaryngology, Shuang Ho Hospital, Taipei Medical University, New Taipei City 23561, Taiwan; 3 International Center for Health Information Technology (ICHIT), College of Medical Science and Technology, Taipei Medical University, Taipei 11031, Taiwan; d610106004@tmu.edu.tw; 4 Research Center of Big Data and Meta-Analysis, Wan Fang Hospital, Taipei Medical University, Taipei 11696, Taiwan; 5 AESOP Technology, Taipei 10596, Taiwan; 6 Clinical Big Data Research Center, Taipei Medical University Hospital, Taipei 11031, Taiwan; 7 Department of Neurosurgery, Shuang Ho Hospital, Taipei Medical University, New Taipei City 23561, Taiwan; 8 Taipei Neuroscience Institute, Taipei Medical University, Taipei 11031, Taiwan

**Keywords:** clinical note, diagnosis codes, medication lists, natural language processing, convolutional neural network

## Abstract

Currently, the International Classification of Diseases (ICD) codes are being used to improve clinical, financial, and administrative performance. Inaccurate ICD coding can lower the quality of care, and delay or prevent reimbursement. However, selecting the appropriate ICD code from a patient’s clinical history is time-consuming and requires expert knowledge. The rapid spread of electronic medical records (EMRs) has generated a large amount of clinical data and provides an opportunity to predict ICD codes using deep learning models. The main objective of this study was to use a deep learning-based natural language processing (NLP) model to accurately predict ICD-10 codes, which could help providers to make better clinical decisions and improve their level of service. We retrospectively collected clinical notes from five outpatient departments (OPD) from one university teaching hospital between January 2016 and December 2016. We applied NLP techniques, including global vectors, word to vectors, and embedding techniques to process the data. The dataset was split into two independent training and testing datasets consisting of 90% and 10% of the entire dataset, respectively. A convolutional neural network (CNN) model was developed, and the performance was measured using the precision, recall, and F-score. A total of 21,953 medical records were collected from 5016 patients. The performance of the CNN model for the five different departments was clinically satisfactory (Precision: 0.50~0.69 and recall: 0.78~0.91). However, the CNN model achieved the best performance for the cardiology department, with a precision of 69%, a recall of 89% and an F-score of 78%. The CNN model for predicting ICD-10 codes provides an opportunity to improve the quality of care. Implementing this model in real-world clinical settings could reduce the manual coding workload, enhance the efficiency of clinical coding, and support physicians in making better clinical decisions.

## 1. Introduction

The International Classification of Diseases (ICD) is a classification system that is widely used by physicians and other healthcare providers for classifying diseases; it includes a wide variety of signs, symptoms, abnormal findings, complaints, and causes of injuries or diseases [1,2,3]. Although the International Statistical Institute (ISI) introduced the first international classification of diseases in 1893 [4], it only gained popularity after 1948 when the World Health Organization (WHO) took responsibility for publishing the ICD and used it to collect health data [5]. The ICD was developed to increase international comparability for the management of morbidity and mortality statistics, reimbursement, and decision support in healthcare [6,7]. The ICD codes have descriptions of diseases or injuries, and each disease has a unique identifier used to code morbidity data from patients’ clinical history. The 10th version of the ICD codes provides better clinical information and consists of more than 70,000 disease codes [8].

In an outpatient setting, physicians usually assign ICD-10 codes for every encounter based on the information from corresponding clinical notes. The manual selection of ICD-10 codes is challenging, time-consuming, and prone to error because the codes show nuanced variations in the specific diagnoses [9]. The selection of inappropriate codes at the time of order could result in improper patient care. Therefore, automatic coding systems have gained people’s interest and a rule-based approached was developed for the automatic assignment of ICD-9 codes [10,11,12]. However, developing methods for the automatic assignment of ICD-10 codes is a labor-intensive, time-consuming, and challenging task due to the high dimensional nature of the problem (over 70,000 codes). Moreover, a large number of rules could make the system complicated even for disease coders. The rapid spread of electronic medical records (EMRs) has generated large amounts of patient data and provides an opportunity to develop an automated tool using deep learning (DL). DL has shown promising outcomes in many domains, especially text classification [13,14,15].

Current approaches to automatically selecting ICD-10 codes have several limitations. The performance of these approaches is not clinically satisfactory. However, our study mainly focused on multi-label text classification from clinical notes in EMRs. We used a top-10 ranking method that can predict the most relevant ICD-10 codes chronologically. Moreover, we showed the coding variance and completeness of ICD-10 codes through the manual review of the false-positive results for each prediction. The prediction of ICD-10 codes from medication lists using CNN-based multi-label classification is a new contribution to this research field. Therefore, our aim was to develop and validate a deep learning model that can assist the physicians in selecting appropriate diagnosis codes at the time that medication is ordered.

## 2. Methods

**Setting, Data Sources, and Study Population:** This study was conducted at a university teaching hospital in Taipei, Taiwan, from 1 January 2016 to 31 December 2016. We retrospectively collected data from the EMRs of this hospital. We considered the clinical notes of several outpatient departments (OPDs); these clinical notes consisted of patient complaints, medications, and diagnosis lists. However, we only utilized drugs (identified by the local hospital drug code and disease history (identified by the International Classification of Diseases (ICD-10 codes) in our study. Patients with incomplete clinical notes were excluded. We included 21,953 medical records from five departments (cardiology, neurology, nephrology, metabolism, and psychology) who visited at least during the study period. A total of 21,953 clinical notes from 5016 patients were considered in the final model. The study was approved by the Taipei Medical University-Joint Institutional Review Board (TMU-JIRB).

**Data Pre-processing:** The clinical notes had different lengths and contained some information that was not relevant to our study outcomes. However, clean and relevant information was needed to develop a clinical notes-based deep learning model. Therefore, we first transformed the raw clinical text to clean text. In the “text cleaning” process, we removed punctuation and numerical values from clinical notes. Moreover, we removed “stop words” such as “a”, “an”, “and”, “for”, “it”, and “the” because they have little predictive value. Stemming, term frequency-inverse document frequency (TF-IDF) vectorization was used for data preprocessing. All preprocessing was conducted using Python Version 3.8, and the Natural Language Toolkit (NLTK) package, version 3.8.

**Figure 1:**
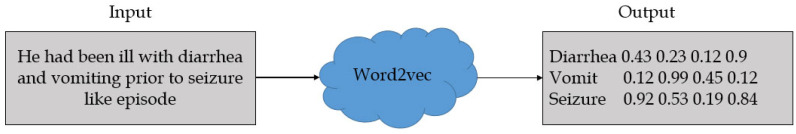
Word2vector process.

**Feature Extraction:** There are different available techniques that can be used to extract information from raw data and to train deep learning models. In this study, we used the Word2vec technique, which helped us to produce a word embedding. This technique can automatically accept text corpus as an input and outputs a vector representation for each word as shown in Figure 1.

**Figure 2:**
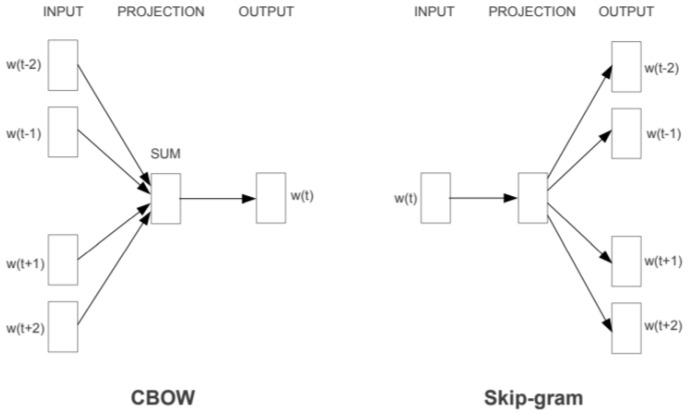
Architecture of CBOW and skip-gram.

The Word2vec algorithm can utilize either a continuous bag-of-words (CBOW) or a skip-gram model to generate a distributed representation of words. The CBOW model predicts the current word from the representation window of context. However, in the skip-gram model, the distributed representation of the input word is utilized to predict the context. A neural word embedding represents a word with numbers. Indeed, word2vec is similar to an autoencoder; it helps to encode each word in a vector. Unlike a restricted Boltzmann machine, word2vec trains words against other words that are next to them in the input corpus. Word2vec works in one of two ways, either utilizing context to predict targeted words, which is known as CBOW, or predict a target context, which is known as skip-gram (Figure 2).

**Figure 3:**
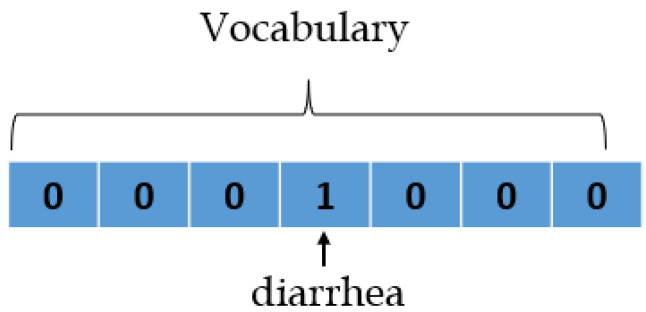
A hot vector.

The skip-gram model utilizes a corpus of text, and it then generates a hot-vector for each word. A hot vector is used to represent a word where the vector is the size of the vocabulary (total number of unique words in the text). All dimensions become 0 except the dimension representing the word that is taken as an input. Figure 3 shows an example of a hot vector.

**Figure 4:**
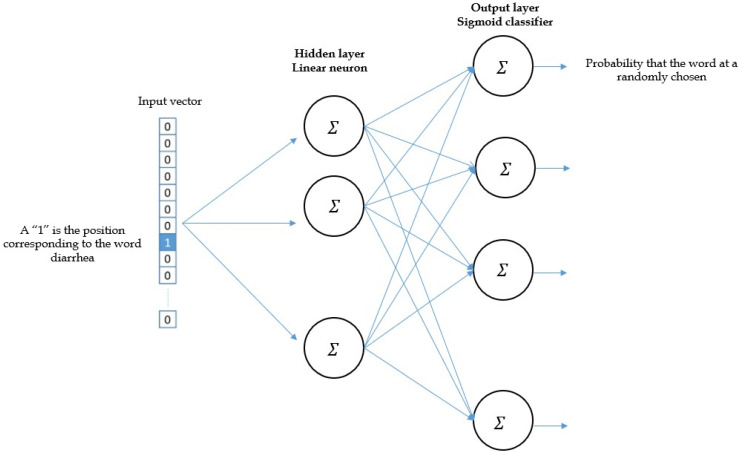
Architecture of neural network.

The hot-vector input is given to neural network with a single hidden layer. In word2vec, a sequence of text is used to convert a distributed representation of words and use a vector with various number of dimensions (e.g., 1000). Each word is then randomly carried various distribution of weights across those elements (Figure 4).

The dimensions of the input vector are used 1 × V, where V is *the number of words in the vocabulary*—that is a one-hot representation of the word. For a single hidden layer, it is V × E dimension, where E is *the size of the word embedding*. The number of features is a hyper-parameter, which is tuned over time. The output from the hidden layer is the dimension 1 × E, and *sigmoid* function is used in this layer. The dimensions of the output layer are 1×V, where each value in the vector represents the probability score of the target word at that position.

**Figure 5:**
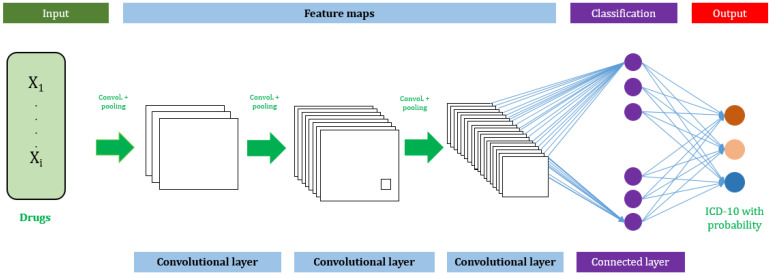
Architecture of the deep-ADCA model.

**Model Development:** We constructed a CNN classification model to predict ICD-10 codes. In the deep learning model, word2vec was first used to generate low-dimensional and dense feature vectors as inputs. We set the dimension of vector generated by word2vec to 128 in training and set the parameter fixed by sample length = 200 Matrix length. In the convolutional layer, the filter window width was fixed (k = 128), and the window sizes were 1, 2, 3, 4, and 5 respectively, to capture different n-gram features. In the maximum pooling layer, the feature map was then extracted for the most significant features for subsequent classification (Figure 5).

**Figure 6:**
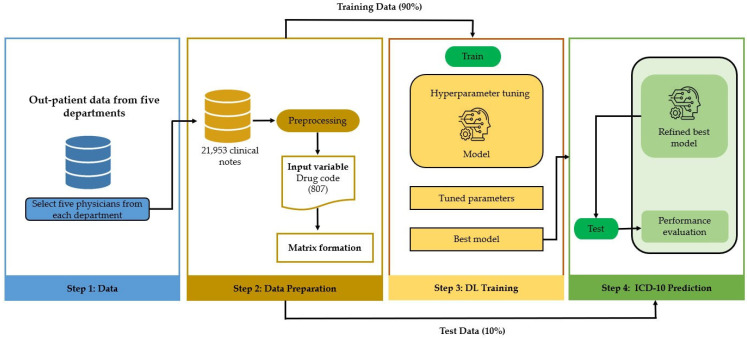
The overall process used in our study.

We considered the task to be a multi-label text classification problem, where the list of medications was used as the input, and the list of ICD-10 codes was the output. We implemented a word embedding word2vector CNN for this multi-label classification task using python and Keras. The sigmoid activation function was used in the final layer of the CNN. A top-10 ranking method was used to obtain diagnoses because this method will likely have smaller prediction errors. The ranking method can identify the best diagnosis chronologically. The model was trained using 90% of the dataset and tested using 10% of the dataset. The overall process is presented in Figure 6. 

**Performance Evaluation:** We assessed the model’s performance using the precision, recall and F-score. The mathematical equations used to calculate the precision, recall and F-score are given below:

***Precision:*** The precision is the ratio of the number of true positives results to the number of all positives results. It measures the model’s accuracy in classifying a sample as positive. Equation (1):

(1) $$Precision=\frac{True\;Positive\;\left(TP\right)}{True\;Postive\;\left(TP\right)+False\;Positive\;\left(FP\right)}$$

***Recall:*** The recall is calculated as the ratio of the number of positive samples that were correctly classified as positive to the total number of positive samples. It helps to measure the model’s ability to detect positive samples. The higher the recall, the more positive samples were detected. Equation (2):

(2) $$Recall=\frac{True\;Positive\;\left(TP\right)}{True\;Positive\;\left(TP\right)+False\;Negative\;\left(FN\right)}$$

***F-score:*** It combines the precision and recall of a classifier into a single metric by taking their harmonic mean. Equation (3):

(3) $$F\text{-}score=\frac{2\times Precision\times Recall}{Precision+Recall}$$

## 3. Results

**Patient Characteristics:** A total of 21,953 clinical notes from 5016 patients (2212 (44.09%) male patients, and 2804 (55.91%) female patients) were included in this study (Table 1). The age range of the patient was between 10 and 101 years. The largest number of clinical notes included were from the psychiatry department, followed by the nephrology and metabolism departments.

**Model Performance**: The performance of the CNN model was assessed using the precision, recall, and F1-score of the model for the testing set; these values are reported in Table 2. The CNN model had the promising discriminative capabilities for the prediction of ICD-10 codes. The CNN model achieved the best performance for the cardiology department, with a precision of 0.69 and recall of 0.91. The precision and recall for the metabolism department were 0.64, and 0.91, respectively.

**Figure 7:**
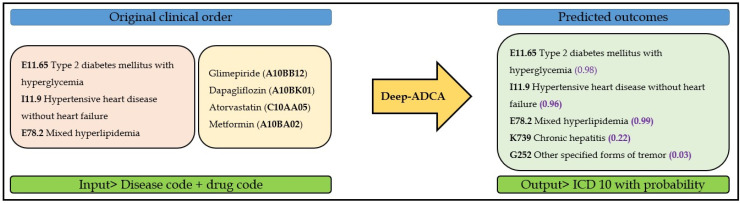
Probabilities of ICD-10 codes predicted from given inputs.

**Performance Evaluation**: After developing and internally validating our CNN model, we evaluated its effectiveness using clinical notes unknown to the model. In the original clinical order, the doctor input three ICD-10 codes and four drugs for the patient. However, our model predicted the correct disease codes based on these variables. Figure 7 shows how our CNN model predicted ICD-10 codes based on simple input variables such as drugs and disease history.

**Figure 8:**
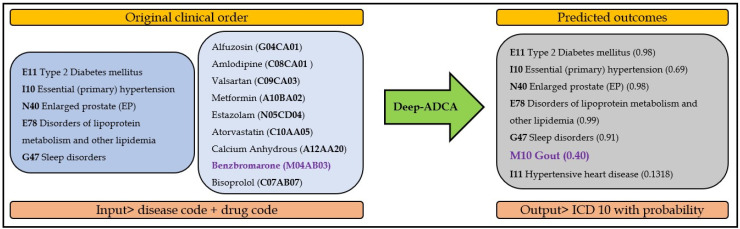
Prediction of missing diagnosis based on input drug.

**Manual Review of Data and Interpretation:** A manual review was also conducted to check the overall appropriateness of our Deep-ADCA model. Our model not only predicts appropriate ICD-10 codes based on the drugs prescribed, but it also shows a missing diagnosis and completes the order accurately. Figure 8 shows that Benzbromarone, an antigout medication, was ordered for the patient; however, the principal diagnosis code gout (M10) was missing in the clinical order. Our model predicted a gout diagnosis based on the antigout medication in the clinical order. Therefore, our model has immense potential to identify non-checked disease from a mix of multiple codes.

## 4. Discussion

**Principal Findings:** In the present study, we aimed to develop a model to automatically predict ICD-10 codes from clinical notes. Using only the medication history, this model achieved good performance in predicting ICD-10 codes. However, the performance of deep-ADCA was best for the cardiology department, followed by the metabolism and psychiatry departments. With this ability, our model can help physicians by providing ICD-10 recommendations at the time that medication is ordered.

**Comparison with Prior Study:** We demonstrated the utility of a deep learning model in ICD-10 coding applications. The automated ICD-10 prediction systems developed in this study can improve the accuracy of diagnosis coding by decreasing the amount of manual coding and coding errors. Since the performance of our model is clinically satisfactory and the model can identify missing diagnoses, using this model can help the physician improve coding accuracy and potentially reduce missing diagnoses and processing times. Many studies have been conducted to evaluate the performance of deep learning models on prediction of ICD-10 codes. However, the majority of these studies used MIMIC discharge summaries to develop the models, and the performances of the models was not satisfactory. Kavuluru et al. [16] evaluated the performance of supervised learning approaches to predict the International Classification of Diseases (ninth revision)—Clinical Modification (ICD-9-CM) codes and obtained a micro-F-score of 0.48. Shi et al. [17] also developed a hierarchical deep learning model using discharge notes from MIMIC, and they were able to automatically assign ICD diagnostic codes given a written diagnosis. Their model achieved an F-score of 0.53 and the area under curve of the receiver operating characteristic of 0.90 for 50 ICD codes. Table 3 shows the performance comparison between previous studies and the current study.

**Strength and Limitations:** Our study has several strengths. First, this is the first study to evaluate the performance of the CNN model on the prediction of ICD-10 codes using only drug histories. Second, our model can predict ICD-10 codes accurately at the time of the drug order, which can help physicians complete their orders efficiently and effectively. Third, our model provides the top 10 diagnosis suggestions based on a probability ranking; therefore, physicians can select any diagnosis on the diagnosis lists, but diagnoses with higher probabilities will be more accurate.

This study also has some limitations that need to be addressed. First, our model was trained using data from a single hospital, which may limit the generalizability of our findings. The performance of our model could vary if the data from other hospitals are used. Second, we used data from only five departments, and the amount of data was not large. The inclusion of other department data might increase the performance of our model. Third, this study has not been validated using an external dataset; further external validation in clinical practice is needed.

**Future perspective:** The proposed prediction model shows the superiority of detecting ICD-10 and identifying the missing diagnosis/es from a mix of multiple codes. Indeed, effective implementation enables physicians in better decision-making and reduces the manual entry of disease codes. As our results are promising, we will use more datasets from various departments and add more features to make it more effective and reliable.

**Conclusion:** In this study, we developed a deep-ADCA model to predict the ICD-10 codes automatically based on medication histories. The performance of our model is clinically satisfactory and better than the performance of models used in previous studies. The findings of this study suggest that a deep learning model trained on a relatively small set of data can be helpful in predicting diagnosis codes accurately. However, external validation is needed before implementation.

## Figures and Tables

**Table 1:** Patient characteristic.

Characteristics		Number (%)
Total Number of Patient		
	Male	2212
	Female	2804
	Age in year, mean (SD), year	60.76 (18.38)
Total number of clinical notes	All departments	21,953
	Cardiology	3668
	Neurology	2762
	Nephrology	5789
	Metabolism	3707
	Psychiatry	6027

**Table 2:** Performance of CNN model for different departments.

Department	Test Cases	No. of ICD-10 Codes	No. of Drugs	Precision	Recall	F-Measure
Cardiology	284	148	145	0.69	0.89	0.78
Metabolism	307	155	136	0.64	0.91	0.75
Psychiatry	475	193	128	0.50	0.87	0.64
Nephrology	432	277	221	0.48	0.84	0.62
Neurology	282	358	177	0.50	0.78	0.61

**Table 3:** The performance comparison between previous studies.

Study	Approach	Dataset	Input	Target	Performance
Xie et al. [18]	Deep learning	MIMIC-III	Diagnosis description	2833 ICD-9 codes	Sensitivity: 0.29 Specificity: 0.33
Huang et al. [19]	Deep learning	MIMIC-III	Discharge summary	10 ICD-9 codes and 10 blocks	F1 score: Full code-0.69, ICD-9 block-0.72
Zeng et al. [20]	Deep learning	MIMIC-III	Discharge summary	6984 ICD-9 codes	F1 score-0.42
Samonte et al. [21]	Deep learning	MIMIC-III	Discharge summary	10 ICD-9 codes	Recall: 0.62, F1-score: 0.67
Hsu et al. [22]	Deep learning	MIMIC-III	Discharge summary	Chapters (19), 50 and 100 ICD-9 codes	Micro F1 score: 0.76 Full code: 0.57 top-50; 0.51-top-10
Gangavarapu et al. [23]	Deep learning	MIMIC-III	Nursing notes	19 Chapters	Accuracy- 0.83
Singaravelan et al. [24]	Deep learning	Medical Center	Subjective component	1871 ICD-19 codes	Recall score: Chapter-0.57, block—0.49, Three-digit code-0.43, Full code—0.45
Our study	Depp learning	Medical Center	Clinical notes	1131 ICD-10 codes	Precision: 0.50~0.69 Recall: 0.78~0.89 F1 score: 0.61~0.78

## Data Availability

Not applicable.
